# First Report of Human Infection Caused by *Aspergillus steynii* and Analysis of Its Whole‐Genome Characteristics

**DOI:** 10.1155/tbed/4093308

**Published:** 2026-06-12

**Authors:** Ruixuan Wang, Jingjing Chen, Na Wu, Min Yang, Ruiying Quan, Nan Xia, Hailong Li, Hua Nian, Ning Jiang, Yunzhuo Chu, Shitong Cheng, Sufei Tian

**Affiliations:** ^1^ National Clinical Research Center for Medical Auxiliary Technology (Laboratory Medicine), Department of Laboratory Medicine, The First Hospital of China Medical University, Shenyang, China, cmu.edu.cn; ^2^ Department of Infectious Diseases, The First Hospital of China Medical University, Shenyang, China, cmu.edu.cn; ^3^ China Medical University—The Queen’s University of Belfast Joint College, China Medical University, Shenyang, China, cmu.edu.cn; ^4^ NHC Key Laboratory of AIDS Prevention and Treatment, The First Hospital of China Medical University, Shenyang, China, cmu.edu.cn

**Keywords:** *Aspergillus steynii*, immunosuppression, multidrug resistance, pulmonary infection, whole-genome sequencing

## Abstract

The incidence of infections caused by rare pathogens has increased in recent years. This necessitates the development of effective diagnostic and therapeutic approaches. In this study, we report the first documented case of *Aspergillus steynii*‐induced pulmonary infection in humans. We evaluated the morphological and molecular characteristics of this fungus to elucidate its role in human infections. A 47‐year‐old woman with immunosuppression after bone marrow transplantation developed symptoms of pulmonary infection. The pathogen was identified as *A. steynii* through culture techniques, microscopy, mass spectrometry, multigene molecular identification, and whole‐genome sequencing. Antifungal susceptibility testing was performed, and comparative genomic analysis was conducted to assess the phylogenetic relationship and genomic characteristics of *A. steynii* and other pathogenic *Aspergillus* species. Genomic analysis revealed a high degree of similarity with other pathogenic *Aspergillus* species. Furthermore, we identified 470 unique gene families primarily associated with ABC transporter pathways linked to multidrug resistance. The strain was sensitive to triazoles and echinocandins but exhibited elevated minimum inhibitory concentrations (MICs) for amphotericin B, flucytosine, and fluconazole. In addition, multiple potential drug resistance genes were identified, indicating the potential for multidrug resistance. The emergence of *A. steynii* in humans poses new clinical challenges and risks, including cross‐species transmission and multidrug resistance. The potential for *A. steynii* infections, particularly in immunosuppressed patients, highlights the importance of early diagnosis and timely intervention to reduce the risk of misdiagnosis or delayed treatment. Thus, our findings have the potential to improve the clinical and differential diagnosis of this infection and facilitate the development of effective therapeutic approaches.

## 1. Introduction

Infections caused by rare pathogens, including bacteria, parasites, and fungi, are increasingly becoming major clinical concerns. This can be attributed to multiple factors, such as the impact of human social behaviors, selective pressures from climate change, and environmental degradation. Infections caused by rare pathogens are often challenging to detect and diagnose using traditional diagnostic tools. This leads to undiagnosed cases, delayed treatments, severe complications, and even death. In India, rare bacterial and fungal infections have been associated with mortality rates as high as 8.4% [1].

The emergence of novel fungal pathogens poses a substantial challenge in clinical medicine. It often leads to severe clinical symptoms, particularly in immunocompromised individuals. The increasing incidence of these infections can be associated with multiple factors, including the overuse of broad‐spectrum antibiotics, prolonged hospital stays, and immunosuppression due to diseases, such as HIV and diabetes, as well as cancer treatment [2–4]. Among these emerging threats, multidrug‐resistant yeast species represent a particularly concerning subset. *Candida auris*, a multidrug‐resistant yeast strain, has become a common nosocomial pathogen. *Candida auris* can colonize hospital surfaces and is resistant to multiple antifungal agents, thus presenting notable challenges in healthcare settings [2, 5]. Given the increasing incidence of these infections, the potential for misdiagnosis using traditional detection methods, and pathogen resistance to existing therapies, clinicians are encouraged to remain vigilant and informed about the evolving landscape of fungal infections to ensure timely and effective patient care [2–6].

The genus *Aspergillus* includes pathogenic and nonpathogenic species that are relatively common causes of human infections. The substantial increase in aspergillosis cases in recent years has made *Aspergillus*‐related infections an emerging focus in clinical microbiology and infectious disease research [7]. The most common pathogenic species are *Aspergillus fumigatus (A. fumigatus*, *Aspergillus flavus* (*A. flavus*), *Aspergillus niger* (*A. niger*), and *Aspergillus terreus* (*A. terreus*) [8, 9]. *A. fumigatus* is the most prevalent cause of pulmonary aspergillosis, followed by *A. flavus* and *A. terreus*. Although *A. niger* and *A. nidulans* are also pathogenic, non‐*fumigatus* aspergilli are often overlooked [10, 11]. Pulmonary aspergillosis accounts for approximately 70% of fungal infection‐related deaths. Clinically, it presents in various forms, including allergic bronchopulmonary aspergillosis, aspergilloma, chronic pulmonary aspergillosis, and invasive aspergillosis. Particularly severe invasive aspergillosis has been observed in immunocompromised individuals [9, 12]. Factors influencing the occurrence and clinical presentation of pulmonary aspergillosis include chronic respiratory diseases, such as asthma, chronic obstructive pulmonary disease, and cystic fibrosis. Similarly, immunosuppression because of conditions, such as COVID‐19, HIV, diabetes, organ transplantation, cancer treatments, and long‐term corticosteroid use, can affect the onset and clinical presentation of this infection [2–4, 13].

We identified and diagnosed a case of pulmonary aspergillosis caused by *A. steynii* in a farmer who underwent bone marrow transplantation in our clinical practice. Prior to this case report, *A. steynii* had been primarily recognized as an agricultural species implicated in ochratoxin A (OTA) contamination of specific food commodities including coffee beans, rice, and fermented beverages [14]. This fungal species exhibits climatic preference, being predominantly isolated from tropical and subtropical regions. To our knowledge, this is the first documented case of *A. steynii* infection in humans. We isolated this pathogen from a clinical specimen determined to be the causative agent of the pulmonary symptoms in the patient. Similar to *A. flavus*, *A. steynii* poses potential risks of toxicity, pathogenicity, and drug resistance. *A. flavus* can infect both plants and humans. Additionally, it can produce aflatoxins that affect growth, reproductive health, liver function, immune responses, and the nervous system. *A. flavus* and *A. terreus* both exhibit elevated minimum inhibitory concentrations (MICs) for amphotericin B and increased resistance to azoles [15], underscoring their clinical importance as crucial fungal pathogens. This raises the possibility of similar concerns regarding *A. steynii*, which warrants heightened clinical awareness. Therefore, we sought to conduct a comprehensive clinical identification and characterization of this pathogenic strain to elucidate its role in human infections. We aimed to increase awareness among clinicians about the potential risks posed by *A. steynii* and emphasize the need for vigilance in its differential diagnosis and recognition in clinical practice.

## 2. Materials and Methods

### 2.1. Strain Selection and Culture

*Aspergillus steynii* CHSY3131 was isolated from the bronchoalveolar lavage (BAL) fluid (BALF) of a 47‐year‐old female patient. Additionally, *A. terreus* and *A. flavus* strains were isolated from the BALF samples of other patients diagnosed with pulmonary aspergillosis. The strains were inoculated onto Sabouraud dextrose agar (SDA) and subcultured twice at 28°C, with each passage lasting 3–4 days. The cultures were subsequently used for morphological observations and sequencing.

### 2.2. Colony Morphological and Microscopic Examination

The strains were subcultured once on SDA at 28°C, followed by inoculation onto potato dextrose agar (PDA), SDA, and Czapek yeast extract agar (CZA). They were then incubated at 28°C for 5 days to observe the colony morphology under different culture conditions. After 5 days on SDA plates, the microscopic morphology of the strains was examined using lactophenol cotton blue staining under a light microscope (40× magnification). Calcofluor white staining (Dynamiker Biotechnology, Tianjin, China) was performed to observe the strains under a fluorescence microscope (60× magnification).

### 2.3. Antifungal Susceptibility Testing

The strains were subcultured on SDA at 28°C and transferred onto PDA for further subculture at 28°C to induce sporulation. Antifungal susceptibility was evaluated using a Yeast One commercial antifungal susceptibility panel (Thermo Fisher Scientific, Waltham, MA, USA) in accordance with the manufacturer’s instructions.

### 2.4. Mass Spectrometry

The colonies were scraped from the surface of the culture medium using a sterile loop to collect fungal hyphae and spores. The fungal material was suspended in sterile EP tubes containing 300 μL high‐performance liquid chromatography‐grade water and 900 μL ethanol solution to extract proteins. The ethanol suspension was centrifuged at 13,000 × *g* for 10 min, and the pellet was dried and resuspended in 25 μL of 70% formic acid (Sigma–Aldrich, Paris, France). Subsequently, 25 μL of 100% acetonitrile (VWR International SAS, Radnor, PA, USA) was added, and the mixture was incubated at 20–25°C for 10 min. The sample was centrifuged at 13,000 × *g* for 2 min before the supernatant was spotted onto a polished steel target plate (MTP 96 target) and air‐dried. Data were acquired using a MALDI‐TOF MS (Vitek, USA) with a mass range of m/z 2000–20,000. Three spectra were collected per target spot.

### 2.5. Conserved Region Sequencing

After two subcultures on SDA at 28°C (3–4 days per passage), the strains were inoculated into a yeast peptone dextrose liquid medium and incubated for 3 days (30°C, 150 rpm). The collected fungal material was then washed with sterile saline. Subsequently, fungal genomic DNA was extracted using a commercial kit (manufacturer specified) with additional wall‐lysing enzymes to facilitate cell wall disruption. PCR amplification of the internal transcribed spacer (ITS), calmodulin (CaM), and β‐tubulin (BenA) was performed under the following cycling conditions: denaturation at 95°C for 30 s, annealing at 58°C for 30 s, and extension at 72°C for 1 min. The following primers were used: ITS, F‐CCG TGT TTC AAG ACG GG, and R‐CTT GGT CAT TTA GAG GAA GTA A; CaM, F‐CCG AGT ACA AGG ARG CCT TC, and R‐CCG ATR GAG GTC ATR ACG TGG; and BenA, F‐GGT AAC CAA ATC GGT GCT GCT TTC, and R‐ACC CTC AGT GTA GTG ACC CTT GGC. The PCR products were sequenced by BGI (Shanghai, China) using Sanger sequencing. The original BALF specimens were directly subjected to metagenomic next‐generation sequencing (mNGS) (BGI).

### 2.6. Phylogenetic Analysis of ITS, CaM, and BenA Sequences and Data Analysis

Phylogenetic analysis was conducted using *A. steynii*. Furthermore, more than 20 additional ITS, CaM, and BenA sequences were downloaded from the NCBI database (https://www.ncbi.nlm.nih.gov/); *A*. sect. Circumdati, *A. terreus*, *A. flavus*, and *A. fumigatus* sequences were used as reference strains. Reference sequences for *A. steynii* were obtained from crops grown in the United States between 2007 and 2016. DNA sequences were processed and aligned using BioEdit software, and phylogenetic analysis was conducted using MEGA 5.0 software [16]. The resulting phylogenetic trees were visualized and refined using the ChiPot software.

### 2.7. De Novo Genome Sequencing

*Aspergillus steynii* CHSY3131 was subjected to whole‐genome shotgun sequencing. Genomic libraries with varying insert sizes were constructed and sequenced using NGS on the Illumina NovaSeq platform. In addition, single‐molecule real‐time (SMRT) sequencing was performed using the PacBio Sequel platform to complement the data from the NGS libraries.

Genomic DNA was extracted using the cetyltrimethylammonium bromide method with minor modifications [17]. DNA concentration, quality, and integrity were then determined using a Qubit Fluorometer (Invitrogen, Carlsbad, CA, USA) and a NanoDrop Spectrophotometer (Thermo Fisher Scientific). Sequencing libraries were generated using the TruSeq DNA Sample Preparation (Illumina, USA) and Template Prep (Pacific Biosciences, USA) kits. Genome sequencing was then performed by the Personal Biotechnology Company (Shanghai, China) using the Pacific Biosciences and Illumina Novaseq platforms. The data obtained through PacBio platform sequencing were then assembled [18, 19] after rectification using the Pilon software [20].

Homologous gene predictions were obtained from the protein sequences of related species using Exonerate (v. 2.2.0) [21]. The predicted genes were integrated using EVidenceModeler v. r2012‐06‐25 [22]. Function annotation was completed through BLAST search against different databases, including the non‐redundant (NR) protein database, Gene Ontology (GO), Kyoto Encyclopedia of Gene and Genomes (KEGG), Swiss‐Prot, Pathogen–Host Interactions (PHI) database, and Transporter Classification Database (TCDB) [23].

### 2.8. Comparative Genomic Analysis

Comparative genomic analysis was conducted to explore genetic similarities and differences between *A. steynii* CHSY3131 and other closely related *Aspergillus* species. Genomic sequences of *A. fumigatus*, *A. flavus*, *A. terreus*, and other strains were retrieved from publicly available databases for comparison. This analysis aimed to identify shared and unique genes, with a particular focus on genes associated with pathogenicity, drug resistance, and environmental adaptation. Bioinformatic tools were used to align the genomes and identify orthologous genes, gene clusters, and variations in metabolic‐ and resistance‐related pathways. The presence of unique genes in *A. steynii* CHSY3131, particularly those involved in multidrug resistance and virulence, was examined to assess their potential impact on pathogenicity and clinical outcomes.

Gene families and single‐copy orthologous genes were analyzed using OrthoFinder v2.5.4 software [24]. Single‐copy homologous genes were selected based on the results of homologous gene cluster analysis for multiple sequence alignments and an alignment quality control system (MAFFT software for sequence alignment, http://mafft.cbrc.jp/alignment/software/) [25]. A phylogenetic tree was constructed using the ML algorithm in FastTree software (http://www.microbesonline.org/fasttree/) [26]. The mcmctree program of PAML software [27] was used to estimate the divergence times among species, and the calibration was based on the divergence time recorded on the TimeTree website [28]. Collinearity analysis was performed using JCVI software (https://github.com/tanghaibao/jcvi) [29].

## 3. Results

### 3.1. Case Description

A 47‐year‐old female patient was diagnosed with acute granulocytic leukemia and received standard chemotherapy at the Hematology Department of the First Hospital of China Medical University on September 28, 2022. The patient had undergone allogeneic hematopoietic stem cell transplantation 7 months prior. Before admission, the patient received decitabine chemotherapy (9 mg/day for 5 days). The patient presented with progressive dyspnea and wheezing upon exertion that lasted for a month upon the admission for chemotherapy.

**Figure 1:**
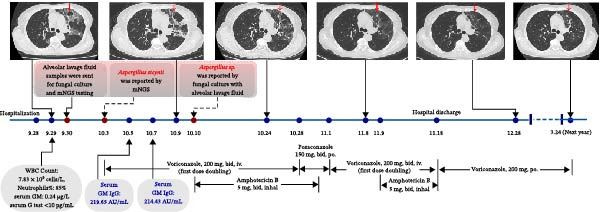
Timeline of clinical course in a patient with *Aspergillus steynii* infection.

The white blood cell count of the patient reached 7.83 × 10^9^ cells/L on September 29, 2022, with 85% neutrophils detected. C‐reactive protein (CRP), interleukin‐6 (IL‐6), and lactate dehydrogenase levels were 73.3 mg/L, 482.96 pg/mL, and 3.5 mmol/L, respectively. A chest CT performed on the same day revealed scattered ground‐glass opacities and nodules in both lungs; a few showed calcification, interstitial changes, and slightly enlarged mediastinal lymph nodes, suggesting a high probability of bilateral inflammatory changes (Figure 1).

The image illustrates the entire course of the treatment of the patient from the detection of *A. steynii* infection to the conclusion of antifungal therapy, including infection‐related blood tests, BALF‐related testing, changes in pulmonary CT lesions, and details of the antifungal treatments administered. Blue represents positive serum test results, whereas red indicates direct culture detection and mNGS identification of *A. steynii*. Red arrows indicate the areas of pulmonary lesions identified on CT. Bid, twice‐daily dosing; iv, intravenous administration; inhal, nebulization therapy; po, oral administration.

The following microbiological findings were reported on September 29, 2022: serum (1, 3)‐β‐D‐glucan was negative (<10 pg/mL); serum galactomannan (GM) antigen was negative (0.24 μg/L). Fungal cultures and mNGS of the BALF were performed. Here, mNGS identified the strain as *A. steynii* (944 reads) on October 3, 2022. GM IgG was 219.65 AU/mL on October 5, 2022, and GM IgG was 214.43 AU/mL on October 7, 2022. The culture yielded *Aspergillus* spp. on October 10, 2022. However, mass spectrometry could not distinguish between *A. ochraceus* and *A. westerdijkiae*. Antifungal susceptibility testing of the isolated strain yielded the following MIC results: anidulafungin MIC = 0.015 μg/mL, micafungin MIC = 0.008 μg/mL, caspofungin MIC = 0.008 μg/mL, 5‐fluorocytosine MIC >64 μg/mL, posaconazole MIC = 0.12 μg/mL, voriconazole MIC = 0.25 μg/mL, itraconazole MIC = 0.12 μg/mL, fluconazole MIC >256 μg/mL, and amphotericin B MIC = 8 μg/mL. The patient’s clinical instability precluded repeat BAL; thus, subsequent microbial isolation and antibiotic susceptibility profiling were unobtainable.

Pulmonary aspergillosis was diagnosed based on these findings. The patient began antifungal treatment with voriconazole (200 mg twice daily with an initial loading dose) on October 3, and continued treatment for 26 days. Amphotericin B nebulization (5 mg twice daily) was added to the regimen on October 10; treatment was continued for 18 days. A follow‐up chest CT on October 24 showed partial resolution of the ground‐glass opacities, indicating improvement. The patient was then switched to oral posaconazole suspension (10 mL, twice daily) for 3 days, beginning on October 28. Voriconazole (200 mg twice daily with an initial loading dose) was resumed on November 1 for an additional 18 days. The patient was then discharged and maintained on oral voriconazole tablets until March 24, 2023. Following 5 months of voriconazole therapy, a repeat chest CT scan performed on March 24, 2023, demonstrated complete resolution of the previously observed ground‐glass opacities. The timeline of the clinical course, diagnosis, and treatment of the patient during hospitalization is shown in Figure 1.

### 3.2. SMRT Sequencing, Assembly, and Annotation

De Novo SMRT sequencing was performed to elucidate the genetic background of the strain. The genome of this strain exhibited the highest match with *A. steynii* (95% identity). Therefore, we identified it as *A. steynii*. The strain was designated CHSY3131, and its genomic map is shown in Supporting Information 1: Figure S1. The total genome length of the strain was 42,601,056,466 bp, and it contained 11,836 genes. The strain belongs to the order Eurotiales, family Aspergillaceae, genus *Aspergillus*, and subgenus Circumdati. Details of the genome assembly and functional annotations are presented in Table 1.

**Table 1:** Genomic assembly and functional annotation of the *Aspergillus steynii* CHSY3131 genome.

Genomic parameter (s)	Value	Database/genomic analysis approach	Count	Percentage (%)
Total length (bp)	42,601,056,466	NR	11,836	98.91
Max length (bp)	396,187	EggNOG	10,962	91.61
GC content (%)	47.24%	KEGG	4211	35.19
Gene number	11836	SwissProt	8552	71.47
Total gene number (bp)	11,966	GO	8185	68.40
Gene/genome (%)	51.5061%	P450	11,723	97.97
Contigs	48	TCDB	2106	17.60
Scaffolds	48	Pfam	8975	75.00
Contigs N50	9736 bp	PHI	3227	26.9
Scaffolds N50	4,833,791 bp			

### 3.3. Comparative Genomic Analysis of CHSY3131 With 30 Clinical Isolates of Pathogenic *Aspergillus* Species

**Figure 2:**
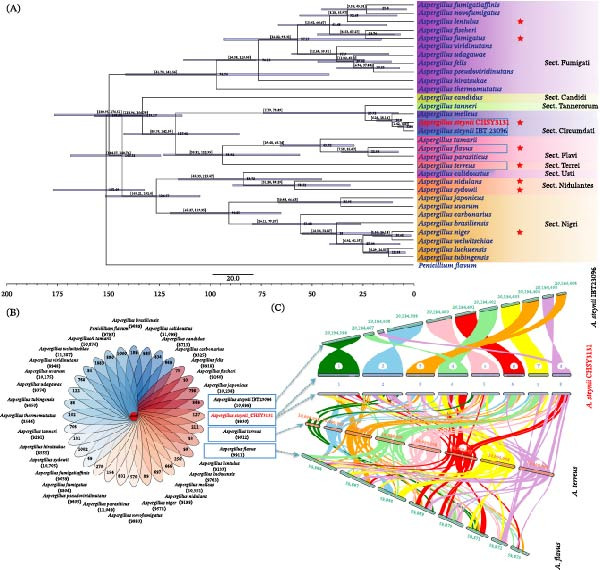
Comparative genomic analysis of CHSY3131 and 30 pathogenic *Aspergillus* species. (A) Phylogenetic tree and divergence time estimation analysis based on single‐copy genes from 32 samples, with *Penicillium flavum* as the outgroup. Asterisks indicate clinically common pathogens. (B) Gene family analysis. (C) Collinearity analysis among four strains: CHSY3131, *A. steynii* IBT 23096, *A. terreus* ATCC 20542, and *A. flavus* NRRL 3357.

As *A. steynii* CHSY3131 is the first reported case of human infection caused by this species, a comparative genomic analysis was conducted to identify the similarities and differences between the genomes of CHSY3131 and those of previously known pathogenic *Aspergillus* species. Genomic information of 30 pathogenic *Aspergillus* species (Supporting Information 2: Data 1) was collected and compared with that of CHSY3131 through evolutionary analysis (Figure 2, Supporting Information 3: Data 2). CHSY3131 was closely related to the clinically common *A. flavus* and *A. terreus* (Figure 2A).

Comparative genomic analysis was performed to further analyze the differences in gene families between CHSY3131 and the 30 pathogenic *Aspergillus* species (Figure 2B). Thirty *Aspergillus* species and CHSY3131 shared 170 gene families, encompassing 400 shared genes. CHSY3131 contained 127 unique gene families. Collinearity analysis was conducted between CHSY3131 and its reference genome, IBT 23096, *A. terreus*, and *A. flavus* (Figure 2C). A significant collinearity was observed between *A. steynii* CHSY3131 and *A. steynii* IBT 23096. Compared to IBT 23096, CHSY3131 showed chromosomal rearrangements within scaffolds 1, 2, 5, and 8. In contrast, each scaffold of CHSY3131 corresponded to multiple scaffolds or chromosomes of *A. flavus* and *A. terreus*. This suggests that breakage or fusion events may have occurred during the evolutionary process.

**Figure 3:**
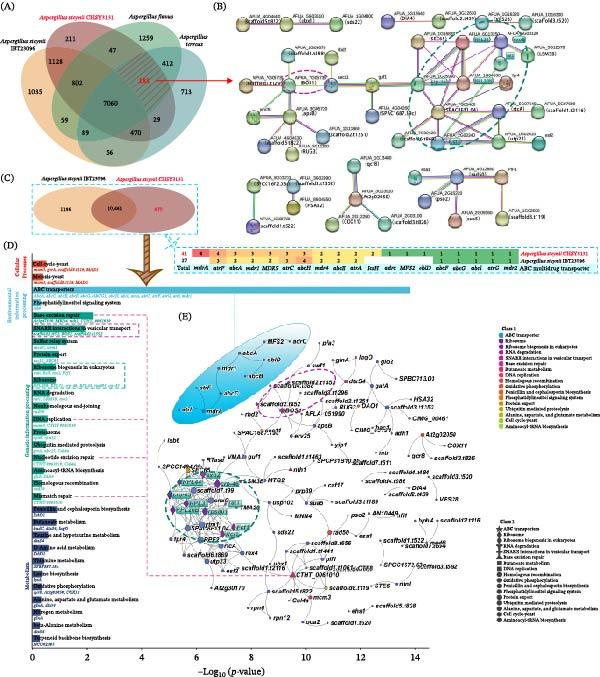
Comparative analysis of CHSY3131. (A) Gene family analysis of four genomes: CHSY3131, *Aspergillus steynii* IBT 23096, *A. terreus*, and *A. flavus*. Red indicates the shared gene families across the CHSY3131, *A. terreus*, and *A. flavus* genomes. (B) PPI analysis of the shared proteins from the CHSY3131, *A. terreus*, and *A. flavus* genomes. In total, 183 gene families were used for PPI analysis; homologous proteins from *A. fumigatus* Af293 were used as reference. Each circle represents a protein, and the lines between circles represent interactions between proteins; different colors indicate varying interaction types. (C) Gene family analysis of CHSY3131 and IBT 23096, with CHSY3131‐specific gene families shown in red. ABC multidrug transporter proteins of the two strains were also shown. (D) KEGG enrichment bar chart of CHSY3131‐specific genes. (E) PPI analysis of CHSY3131‐specific genes. The pink and green dashed circles represent two distinct gene‐enriched clusters.

Comparison of genes from the four genomes revealed 183 shared gene families (Figure 3A, Supporting Information 4: Data 3). Protein–protein interaction (PPI) network analysis revealed two major clusters: ribosomal proteins (RPL20B, RPL31, rat1, and exo2) and SNARE interaction in the vesicular transport signaling pathway (BOS1) (Figure 3B). The detailed protein analysis results are shown in Supporting Information 2: Data 1, Supporting Information 3: Data 2, Supporting Information 4: Data 3.

### 3.4. Comparison of CHSY3131 and the Reference Genome

We conducted a comparative analysis of CHSY3131 and the *A. steynii* IBT23096 reference genome to further elucidate the differences between the genomes of CHSY3131 and previously isolated plant‐derived strains. CHSY3131 harbored 470 unique gene families (Figure 3C). Comparative genomic analysis revealed that strain CHSY3131 encodes 41 unique ABC transporters, contrasting with the 27 identified in reference strain IBT23096. GO enrichment analysis showed that these unique gene families were significantly enriched in membrane transport pathways, including multiple copies of azole‐resistant genes, such as *mtr*, *mdrA*, and *MFS2* (Supporting Information 1: Figure S2A). KEGG enrichment analysis revealed significant enrichment of these gene families in the ABC multidrug transporter pathway, base excision repair, and SNARE interactions in the vesicular transport signaling pathway (*p* < 0.05) (Figure 3D, Supporting Information 1: Figure S2B). Annotation of 470 unique gene families (including 566 genes) indicated that the ABC transporter pathway includes multiple copies of azole resistance‐related genes, such as *abcC*, *abcH*, *abcG*, *artA*, *artF*, *artG*, and *artI* (Figure 3D). The base excision repair pathway also included the DNA glycosylase gene (*MBD4*) derived from the human host.

Notably, PPI analysis of the 470 unique gene families identified four protein‐enriched clusters corresponding to the aggregation of ABC multidrug transporters, genes involved in mismatch repair and modification, SNARE interactions in vesicular transport, and ribosome biogenesis (Figure 3E).

### 3.5. Morphological Comparison

Genomic analyses showed that the affinity of CHSY3131 is similar to that of the common pathogens *A. terreus* and *A. flavus*. Therefore, we compared the colony morphology and strain micromorphology of the three species.

**Figure 4:**
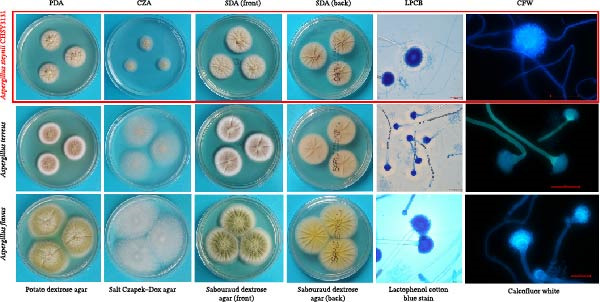
Morphological comparison of CHSY3131 with *Aspergillus terreus* and *A. flavus*. The strains were inoculated onto PDA, CZA, and SDA plates and incubated at 28°C for 5 days. Morphology was microscopically observed under 40× magnification using lactophenol cotton blue staining and under 60× magnification using Calcofluor white fluorescence staining.

CHSY3131, *A. terreus*, and *A. flavus* were cultured on PDA, SDA, and CZA, respectively, at 28°C for 5 days (Figure 4). *Aspergillus steynii* grew relatively slowly on PDA and CZA media. On the SDA medium, *A. flavus* colonies had a dense wooly or cotton‐wooly texture with radial grooves on the front side. The colony color gradually changed from yellow to yellow‐green or gray‐green, and the reverse of the colony was yellowish‐white or brownish. In addition, it was flat or with radial to irregular grooves. The *A. terreus* colony frontal texture was velvety. The center was floccose, with radial or inconspicuous grooves. The frontal color was cinnamon or yellowish brown, whereas the reverse side was yellow. *Aspergillus steynii* colonies were white to yellow. Their central texture was fluffy flocculent with clear radial grooves. Spores were produced densely in the center of the colony. The reverse color was light brown or grayish yellow, similar to that of *A. terreus*.

**Figure 5:**
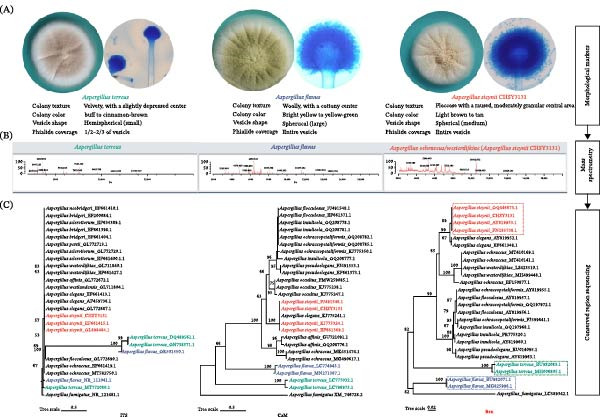
Comparative analysis of protein mass spectrometry and molecular identification for three fungal species. (A) Colony morphology and microscopic morphology of *A. terreus*, *A. flavus*, and CHSY3131. The strains were inoculated onto SDA plates and incubated at 28°C for 5 days. (B) Protein mass spectra for three *Aspergillus* species. (C) Phylogenetic trees based on molecular identification of the ITS, CaM, and BenA regions for three *Aspergillus* species and closely related strains. *Aspergillus fumigatus* was used as an outgroup reference. Red, blue, and green represent *A. steynii*, *A. flavus*, and *A. terreus*, respectively. Dashed lines mark identical *Aspergillus* species, with taxonomic identifications labeled on the figure’s right side.

*Aspergillus steynii* conidial heads radiated slightly larger than those of *A. terrestris* and *A. flavus* under microscopy. The conidiophores of *A. steynii* were biseriate and produced dense sporulation at the colony center (Figures 4 and 5A). In contrast, *A. terreus* and *A. flavus* had conidiophores that radiated in one direction or in a semicircle. The conidiophores of *A. terreus* were short and smooth, the phialides were in two rows and compact columnar, and the conidia were markedly small. *A. terreus* can be distinguished from other *Aspergillus* spp. based on the production of aleuroconidia. The conidiophores of *A. flavus* were variable in length, rough, pitted, and spiny; the phialides were single and double, covered the entire vesicle, and pointed in all directions.

### 3.6. Strain Identification

Protein mass spectrometry is commonly used to identify fungal species in clinical laboratories. However, mass spectrometry did not accurately identify CHSY3131. CHSY3131 was incorrectly identified as *Aspergillus ochraceus* (50% probability) or *Aspergillus westerdijkiae* (50% probability) based on protein abundance matching (Figure 5B). Conversely, mass spectrometry successfully identified *A. terreus* and *A. flavus* (Figure 5B).

Molecular biology techniques involving the amplification and sequencing of conserved regions (ITS, CaM, and BenA) are used to distinguish common clinical *Aspergillus* species. *Aspergillus steynii*, *A. terreus*, and *A. flavus* could not be differentiated solely via ITS sequencing. Although CaM sequencing can distinguish *A. steynii* from *A. terreus* and *A. flavus*, it cannot differentiate between *A. steynii* and *A. elegans*. Only BenA sequencing effectively identified *A. steynii* (Figure 5B,C). These results suggest that *A. steynii* is prone to misidentification, similar to that of other *Aspergillus* species.

The distinguishing characteristics of the three *Aspergillus* species are summarized in Figure 5 and Supporting Information 5: Table 2 to provide a clinical reference differential diagnosis.

## 4. Discussion

To the best of our knowledge, this study is the first to report a case of human infection caused by *A. steynii*, which led to pulmonary infection‐related symptoms in the host. The patient exhibited multiple infection risk factors, including exposure to decaying crops (as she worked as a farmer), undergoing allogeneic hematopoietic stem cell transplantation 7 months prior, receiving regular chemotherapy, and being immunosuppressed. Following the infection, the patient presented with dyspnea; an increased neutrophil percentage; elevated levels of CRP, IL‐6, and lactate dehydrogenase; and scattered ground‐glass opacities and nodules in both lungs, some showing calcifications. Additionally, the peripheral blood white cell count of the patient was normal, and only the serum GM IgG test was consistently positive twice. This suggests that *A. steynii* infection may not cause a significant increase in GM IgM in cases where peripheral white cell counts are normal. Moreover, positive GM IgG results may indicate *A. steynii*‐induced pulmonary infection.

*Aspergillus steynii* has only been reported in the context of agricultural food biosafety, with no prior cases of human infection. Colony morphology and identification using clinical laboratory methods can easily result in misidentification of other *Aspergillus* species, in turn leading to misdiagnosis or missed diagnoses. For example, mass spectrometry can misidentify *A. steynii* as *A. ochraceus* (50% probability) or *A. westerdijkiae* (50% probability). In the present study, *ITS* sequencing could not distinguish between *A. steynii*, *A. terreus*, and *A. flavus*, whereas *CaM* sequencing could not differentiate *A. steynii* from *A. elegans*. Although BenA sequencing and mNGS can accurately identify *A. steynii*, a risk of misdiagnosis or delayed diagnosis remains owing to the lack of awareness of its potential to infect humans. In this study, we comprehensively evaluated this pathogenic strain and summarized its key clinical and morphological features (Supporting Information 5: Table 2). The screening for *A. steynii* can be initially performed by observing its distinctive colony morphology (ocher‐yellow pigmentation with a granular texture) and microscopic characteristics (radiate conidial heads). If mass spectrometry identifies *A*. *ochraceus*, further differentiation from *A. steynii* should be pursued. Definitive confirmation requires BenA gene sequencing as the gold‐standard diagnostic method. Our findings may provide a more in‐depth understanding of *A. steynii* among clinicians and ensure effective care for patients with underlying risk factors, such as crop exposure and immunosuppression. Increased awareness of the potential infection risk of *A. steynii* may ensure vigilance in the differential diagnosis of infections caused by this pathogen.

In addition to the direct risk of infection, *A. steynii* is a common crop contaminant that can produce OTA [30]. OTA has nephrotoxic, hepatotoxic, teratogenic, neurotoxic, and genotoxic properties. Furthermore, it is carcinogenic [31]. Although it remains unclear whether *A. steynii* can produce OTA in an infected human host and trigger toxic reactions, this possibility raises concerns about the potential risk of organ damage following infection. Therefore, clinicians should take this into consideration as the early detection and accurate identification of this fungus may help alleviate infectious symptoms and prevent organ damage in infected individuals.

To further investigate the genetic background of *A. steynii* strain CHSY3131, we compared the CHSY3131 genome to those of the plant‐infecting *A. steynii* IBT 23096 and 30 clinically common pathogenic *Aspergillus* species. Chromosomal rearrangements were observed in scaffolds 1, 2, 5, and 8 in CHSY3131 compared to those in IBT 23096. Phylogenetically, CHSY3131 was closely related to the clinically prevalent *A. flavus* and *A. terreus* (Figure 2A). These results suggest that multiple breakage or fusion events have occurred during evolution, enabling *A. steynii* to gradually acquire the ability to infect humans from its original role as a plant pathogen. Further analysis identified 470 unique gene families in CHSY3131 compared to IBT 23096, with significant enrichment in pathways associated with ABC multidrug transporters, mismatch repair and modification, SNARE interactions in vesicular transport, and ribosome biogenesis (Figure 3E). Fungi exhibit considerable genomic variation, even within the same species [32]. Our results suggest that the expansion of resistance‐related, genome modification and splicing, and ribosomal and energy‐related genes may facilitate the evolution of pathogenic fungi, ultimately conferring the ability to survive in human hosts.

ABC transporters in fungi are known to mediate drug efflux and secondary metabolite secretion [33]. However, significant knowledge gaps persist regarding their functional roles in *Aspergillus* spp. Comparative genomic analysis revealed that *A. steynii* CHSY3131 encodes a greater number of ABC transporters than the plant‐associated strain IBT23096. We hypothesize that this expansion may contribute to environmental adaptation, while the potential overexpression of these transporters could additionally confer azole resistance. The CHSY3131‐specific genes identified in this study include multiple copies of azole resistance genes, such as *mtr*, *mdrA*, and *MFS2* (Supporting Information 1: Figure S2A). Additionally, the ABC multidrug transporter pathway contained multiple copies of azole resistance‐related genes, such as *abcA* (*mdr1*), *abcC*, *abcF*, *abcH*, *abcG1*, *atrA*, *atrF*, and *mtr* (Figure 3D), as well as a DNA glycosylase gene (*MBD4*) derived from the human host. ABC multidrug transporters are important for azole resistance and play a crucial role in multidrug‐resistant *Candida auris*. For example, *mdr1* contributes to itraconazole resistance in *Aspergillus* [34, 35]; *abcG1* is involved in azole resistance [36, 37]; and *AbcC* and *AbcF* contribute to itraconazole and posaconazole resistance [38]. Although antifungal susceptibility testing demonstrated low MIC values of CHSY3131 to triazoles and echinocandins, genomic alignment of its cyp51A and FSK genes (fks1/fks2) with those of *A. fumigatus* revealed no canonical mutations associated with triazole (e.g., TR_34_/L98H) or echinocandin (e.g., FKS1 S678P) resistance [39, 40]. Notably, voriconazole has been shown to rapidly induce a surge in the ABC transporter expression in *Aspergillus* species [41]. This prompt upregulation strongly suggests that CHSY3131 carries an intrinsic risk of developing acquired resistance during antifungal therapy. However, this patient required an extended 5‐month treatment course before achieving significant radiological improvement—substantially longer than conventional therapy durations for clinically common *Aspergillus* species (e.g., *A. fumigatus* and *A. flavus*; typically 6–12 weeks) and exceeding reported median timelines for complete radiological resolution (average 80 days) [42, 43]. This abnormally prolonged treatment course may suggest: (1) progressive MIC increases below conventional resistance thresholds during therapy or (2) partial activation of ABC transporter systems under drug selection pressure, resulting in reduced treatment efficacy. Unfortunately, due to the patient’s deteriorating clinical condition, bronchoscopy with BAL could not be repeated to obtain follow‐up specimens for culture and antifungal susceptibility testing. Given these findings, we strongly recommend enhanced mycological surveillance—including sequential fungal identification and antifungal susceptibility testing—for *A. steynii* infections. Particular attention should be paid to potential voriconazole resistance development during treatment, enabling timely therapeutic adjustments to optimize clinical outcomes. In addition, host body temperature can induce resistance and virulence against first‐line antifungal drugs in novel fungal pathogens [44]. Based on the above, the presence of multiple copies of these resistance genes suggests a high degree of phenotypic variation, plasticity, and differentiation capacity in *A. steynii*, indicating a potential risk of multidrug resistance in strain CHSY3131.

In the case reported in this study, CHSY3131 showed high MICs for 5‐fluorocytosine, fluconazole, and amphotericin B. Further, it exhibited genetic similarity to *A. terreus* and *A. flavus*, which also have elevated MICs for amphotericin B. In addition, abnormal catalase expression has been linked to the innate resistance of *A. terreus* to amphotericin B [45]. Therefore, we hypothesized that catalase may also contribute to the elevated MIC of amphotericin B in *A. steynii*. Taken together, our findings suggest that *A. steynii* may develop elevated MICs to both triazoles and echinocandins during infection, positioning it as a potential emerging multidrug‐resistant fungal pathogen akin to *Candida auris*.

Infections caused by *A. flavus* and *A. terreus*, which are phylogenetically closest to CHSY3131, have increased in recent years. *A. flavus* induces a stronger inflammatory response [46] than *A. fumigatus*, which has been listed as a crucial threat fungus by the World Health Organization. In addition, *A. flavus* infections have shorter survival times and lower 30‐day survival rates [47], whereas *A. terreus* often results in a longer disease course [11]. Similar to these species, *A. steynii* is transmitted from crops to humans and can produce mycotoxins. This suggests that clinicians should remain vigilant regarding the emergence of *A. steynii* pulmonary infections and the potential for increased infection rates, severe disease progression, and extended disease courses.

The first *A. steynii* pulmonary infection case reported in this study further raises concerns regarding cross‐species transmission from crops to humans and the acquisition of environmental resistance. While *A. steynii* has primarily been associated with tropical and subtropical climates based on existing phytopathological reports (citations), our case represents the first documented human infection in a temperate zone (Liaoning, China). This geographical expansion may reflect ecological adaptations potentially influenced by climate change, warranting vigilance for emerging fungal pathogens in nonendemic regions. Climate change is a global concern, with temperatures rising by 3–5°C on average in Europe, accompanied by droughts, rainfall, flooding, and shifts in crop growth cycles. Similar changes have been observed in other crop‐growing regions of Asia and America. These warmer climates may increase the potential for environmental fungi to become clinical pathogens. Climatic conditions, along with poor production and storage practices in developing countries where OTA contamination is severe, may further promote fungal transmission from crops to humans [48]. Agricultural fungicides, particularly azoles commonly used in crop production, can induce resistance mutations in environmental *Aspergillus* species, leading to cross‐resistance to medical triazoles [49]. A 2017 study of azole‐resistant *A. fumigatus* in China revealed regional differences in resistance, with some mutations attributed to exposure to environmental azole fungicides rather than patient treatment [50]. Furthermore, azole fungicides used in crop fermentation may drive triazole resistance in industrially used *Aspergillus* strains [51]. These studies demonstrate the risk of azole‐resistant invasive aspergillosis among patients in resistant regions, which significantly complicates the diagnosis and treatment of clinical *Aspergillus* infections [52]. Therefore, we recommend that agricultural and health authorities closely monitor fungal infections and resistance among agricultural workers, with particular attention paid to their immune status to prevent infections similar to those observed in the present study.

This study had some limitations. Our findings are limited to a single case. Therefore, they cannot completely represent the pathogenic diversity or resistance profile of the entire species. These findings cannot fully capture the pathogenic diversity or antifungal resistance spectrum of *A. steynii* as a species. Moving forward, we will systematically collect clinical strains and associated metadata, enabling comprehensive genomic and phenotypic characterization of this emerging pathogen. The absence of complete genomic information from other host‐derived strains also limits the effective evaluation and confirmation of the SNP data for this strain. Thus, future studies are required to review previous cases that may have been misdiagnosed or undetected as *A. steynii*, as well as to further investigate the virulence and infectivity of *A. steynii* strains that infect humans. This includes exploring virulence in animal models, adaptation to hypoxia, growth under chemical stress, nutrient heterogeneity, and induction of host inflammatory mediators to elucidate the infection mechanisms of this pathogenic fungus and provide a reliable basis for the clinical diagnosis and treatment of *A. steynii* infections.

## 5. Conclusion

To the best of our knowledge, this is the first study to report a case of *A. steynii*‐induced pulmonary infection in humans, particularly in an immunosuppressed patient. We comprehensively evaluated this pathogen using a combination of culture, morphological observations, molecular biology analyses, and whole‐genome sequencing. Our findings highlight the importance of understanding this fungal strain in a clinical setting, particularly in patients with risk factors, such as crop exposure and immunosuppression. Clinicians should consider these risk factors for *A. steynii* infection and remain vigilant during differential diagnosis of infections caused by this pathogen to avoid misdiagnosis or missed diagnoses. We also recommend close monitoring of the infection dynamics of this fungus as it poses notable challenges to clinical practice, including rising infection rates, severe diseases, prolonged disease courses, and the potential for multidrug resistance.

## Author Contributions

Ruixuan Wang, Jingjing Chen, Na Wu, Min Yang, Ruiying Quan, and Hailong Li have made substantial contributions to conception and design, or acquisition of data, or analysis and interpretation of data. Hua Nian, Ning Jiang, and Yunzhuo Chu have been involved in drafting the manuscript.

## Funding

This study was supported by the Noncommunicable Chronic Diseases‐National Science and Technology Major Project (Grants 2024ZD0533100 and 2024ZD0533106), the National Key Research and Development Program of China under Grant 2021YFC2300400, and the National Natural Science Foundation of China under Grant 82202547.

## Disclosure

Sufei Tian and Shitong Cheng have given final approval of the version to be published.

## Ethics Statement

The medical history of the enrolled patient was obtained from the Hospital Information System (HIS) system of the First Hospital of China Medical University and assessed by clinicians. This study was reviewed and approved by the Ethics Review Committee of the First Hospital of China Medical University (ERC Number 2024‐758) in accordance with the principles of the Declaration of Helsinki.

## Conflicts of Interest

The authors declare no conflicts of interest.

## Supporting Information

Additional supporting information can be found online in the Supporting Information section.

## Supporting information


**Supporting Information 1** Figure S1: De novo third‐generation sequencing genome map of *Aspergillus steynii* CHSY3131. Figure S2: Enrichment analysis of CHSY3131‐specific genes. Table S1: Genomic information on 30 pathogenic *Aspergillus* spp.

**Supporting Information 2** Data 1: Gene annotation all_cxj1117_swiss_fmt0_result.

**Supporting Information 3** Data 2: Data for Figure2 ortho2 species.

**Supporting Information 4** Data 3: Data for Figure3 gene annotation.

**Supporting Information 5** Table S2: Summary of differential characteristics of three *Aspergillus* strains.

## Data Availability

The raw sequence data reported in this paper have been deposited in the Genome Sequence Archive (Genomics, Proteomics & Bioinformatics 2021) in the National Genomics Data Center (Nucleic Acids Res 2022), China National Center for Bioinformation/Beijing Institute of Genomics, Chinese Academy of Sciences (GSA: CRA016499 and CRA016500) that are publicly accessible at https://ngdc.cncb.ac.cn/gsa. The processed data, such as gene annotation results, reported in this paper, were also provided as supporting data.
