# Supplementary material for: First Report of Human Infection Caused by Aspergillus steynii and Analysis of Its Whole‐Genome Characteristics
Source: Transbound Emerg Dis. 2026 Jun 12;2026:4093308. doi: 10.1155/tbed/4093308 (PMC13263534; doi:10.1155/tbed/4093308)
Supplement: Supplementary file 1 — Supporting Information 1 Figure S1: De novo third‐generation sequencing genome map of Aspergillus steynii CHSY3131. Figure S2: Enrichment analysis of CHSY3131‐specific genes. Table S1: Genomic information on 30 pathogenic Aspergillus spp. [file TBED-2026-4093308-s001.docx]

**Supplementary information**


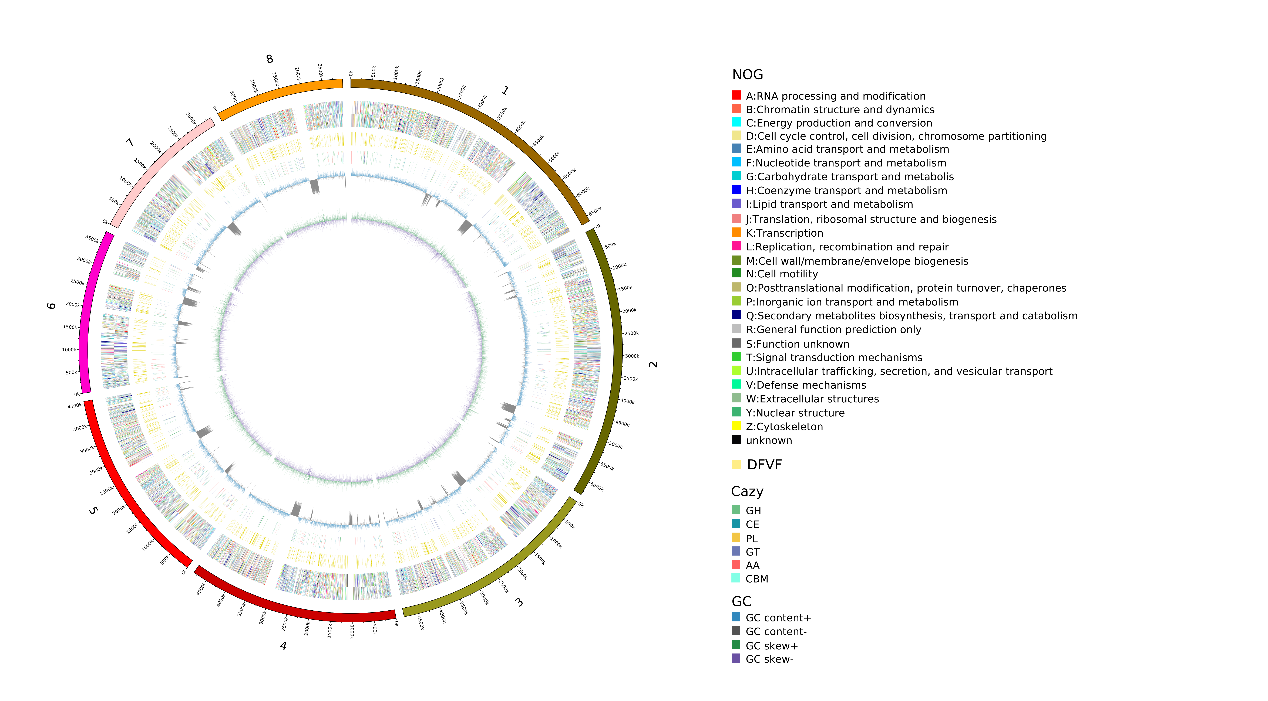


Supplementary Figure 1. De novo third-generation sequencing genome map of *Aspergillus steynii* CHSY3131.
The genome annotation analysis is based on gene prediction results, arranged from inner to outer circles:
1st circle: GC skew;
2nd circle: G + C content;
3rd circle: Carbohydrate-Active enZYmes (CAZy);
4th circle: Database of Fungal Virulence Factors (DFVF);
5th circle: CDS corresponding to each COG on the negative strand;
6th circle: CDS corresponding to each COG on the positive strand;
7th circle: scale.

Supplementary Figure 2. Enrichment analysis of CHSY3131-specific genes.
**A.** GO enrichment analysis: CC, cellular component; MF, molecular function; BP, biological process. **B.** KEGG enrichment bubble chart. Red indicates p < 0.05.

Supplementary Table 1. Genomic information on 30 pathogenic Aspergillus spp.

| **Genomic information on 30 pathogenic Aspergillus spp.** | | |
| --- | --- | --- |
| **Organism name** | **Name** | **GenBank assembly accession** |
| Aspergillus steynii | IBT 23096 | GCA_002849105.1 |
| Aspergillus melleus | CBS 546.65 | GCA_016097325.1 |
| Aspergillus fumigatus | Af293 | GCA_000002655.1 |
| Aspergillus lentulus | IFM 58399 | GCA_010724455.1 |
| Aspergillus calidoustus | / | GCA_001511075.1 |
| Aspergillus flavus | [NRRL3357](https://www.ncbi.nlm.nih.gov/datasets/taxonomy/332952) | GCA_014117465.1 |
| Aspergillus tamarii | CBS 117626 | GCA_009193485.1 |
| Aspergillus parasiticus | CBS 117618 | GCA_009176385.1 |
| Aspergillus terreus | ATCC 20542 | GCA_016808415.1 |
| Aspergillus nidulans | FGSC A4 | GCA_000011425.1 |
| Aspergillus niger | IFM 62618 | GCA_027923985.1 |
| Aspergillus felis | CNM-CM7691 | GCA_014281915.1 |
| Aspergillus tubingensis | IFM 61612 | GCA_027923925.1 |
| Aspergillus sydowii | CBS 593.65 | GCF_001890705.1 |
| Aspergillus candidus | CBS 102.13 | GCA_002847045.1 |
| Aspergillus brasiliensis | IFM 66951 | GCA_027924065.1 |
| Aspergillus fischeri | NRRL 181 | GCA_000149645.4 |
| Aspergillus udagawae | IFM51744 | GCA_010724255.1 |
| Aspergillus carbonarius | ITEM 5010 | GCA_001990825.1 |
| Aspergillus japonicus | CBS 114.51 | GCA_003184785.1 |
| Aspergillus thermomutatus | HMR AF 39 | GCA_002237265.2 |
| Aspergillus viridinutans | IFM 47045 | GCF_018404265.1 |
| Aspergillus tanneri | NIH1004 | GCA_003426965.1 |
| Aspergillus welwitschiae | CBS 139.54b | GCF_003344945.1 |
| Aspergillus fumigatiaffinis | CNM-CM6805 | GCA_012656285.1 |
| Aspergillus hiratsukae | CNM-CM5793 | GCA_014281905.1 |
| Aspergillus luchuensis | RIB2601 | GCA_016865315.1 |
| Aspergillus pseudoviridinutans | IFM 55266 | GCA_018340605.1 |
| Aspergillus uvarum | CBS 121591 | GCF_003184745.1 |
| Aspergillus novofumigatus | IBT 16806 | GCA_002847465.1 |
